# The association of hematological inflammatory markers and psychological function in COVID‐19 patients: A cross‐sectional study

**DOI:** 10.14814/phy2.15889

**Published:** 2023-12-20

**Authors:** Zahra Khorasanchi, Mohammad Rashidmayvan, Elahe Hasanzadeh, Mohammad Reza Shadmand Foumani Moghadam, Nafise Afkhami, Parisa Asadiyan‐Sohan, Mohammad Vahedi Fard, Kimia Mohammadhasani, Naiemeh Varaste, Payam Sharifan, Gordon Ferns, Majid Ghayour Mobarhan

**Affiliations:** ^1^ Department of Nutrition, School of Medicine Mashhad University of Medical Sciences Mashhad Iran; ^2^ Student Research Committee, Faculty of Medicine Mashhad University of Medical Sciences Mashhad Iran; ^3^ International UNESCO Center for Health Related Basic Sciences and Human Nutrition, Department of Nutrition, Faculty of Medicine Mashhad University of Medical Sciences Mashhad Iran; ^4^ Nutrition Sciences Varastegan Institute for Medical Sciences Mashhad Iran; ^5^ Departments of Biology, Faculty of Sciences, Mashhad Branch Islamic Azad University Mashhad Iran; ^6^ Department of Nutrition, Food Sciences and Clinical Biochemistry, School of Medicine, Social Determinants of Health Research Center Gonabad University of Medical Science Gonabad Iran; ^7^ Division of Medical Education Brighton and Sussex Medical School Brighton UK

**Keywords:** anxiety, COVID‐19, depression, hematologic tests, psychological, stress

## Abstract

Mental health disorders are linked to systemic inflammation. Due to high inflammation and mental health disorders in COVID‐19 patients, we aimed to investigate the relationship between blood inflammatory markers such as red cell distribution width to platelet ratio (RPR), platelet‐lymphocyte ratio (PLR), neutrophil/lymphocyte ratio (NLR), red cell distribution width (RDW), white blood cell (WBC), and psychological function in COVID‐19 patients. In the current cross‐sectional study, neuro‐psychological function, and a complete blood count (CBC) were measured on 120 COVID‐19 patients aged >30 years from the Imam Reza Hospital in Mashhad, Iran. Our results showed that anxiety related to MCHC (mean ± SD: 32.71 ± 1.68, *p* < 0.05), WBC (mean ± SD: 12.23 ± 5.43, *p* < 0.05), and PLR (median (IQR): 28.72 (15.88–41.31), *p* < 0.05) significantly. In the stress subgroup, only RPR was associated with stress (*p* < 0.05). Linear regression between hematological parameters and psychological score indicated that RDW and PLR had a significantly positive association with depression (*β* = 0.086; *p* = 0.045 and *β* = 1.326; *p* = 0.016, respectively) and anxiety scores (*β* = 0.100; *p* = 0.038 and *β* = 1.356; *p* = 0.010, respectively). Moreover, a positive correlation was found between PLR and stress (*β* = 1.102; *p* = 0.012). This study showed a positive association between depression/anxiety/stress symptoms and levels of hematological inflammatory markers including PLR and RDW. The findings of this study provide novel insights into mental health and physiological markers, underscoring the potential influence of inflammation on mood disorders. Our findings offer exciting prospects for future research and may lead to innovative approaches in the management and treatment of depression, anxiety, and stress.

## 1 INTRODUCTION

The 2019 coronavirus (COVID‐19) pandemic, has affected more than 170 million individuals resulting in 3.5 million deaths globally by May 2021 (Fine et al., 2011). In addition to the impact on physical health, has taken a toll on mental health as a result of fear of illness and hospitalization, and social distancing efforts may have a unique and significant influence on mental health. Given the significance of stress in the etiology of anxiety and depressive disorders, the prevalence of these diseases may rise due to intensified stress related to COVID‐19. Previous studies have reported on the mental health of COVID‐19 hospitalized patients during the epidemic, while preliminary research suggests a potential link between these symptoms and disorders and a more severe progression of COVID‐19 (Dergaa et al., 2022; Varma et al., 2021). In this context, it is essential to investigate the prevalence and severity of anxiety and depression in COVID‐19‐infected patients (Khorasanchi et al., 2023; Reger et al., 2020).

In anxiety and related disorders, central and peripheral immune system cells release cytokines and cause inflammation in response to increased stress (Michopoulos et al., 2017). Inflammation and anxiety disorders are linked, and hematological parameters are also thought to be useful markers in the follow‐up of patients with anxiety disorders (Uzun & Akinci, 2021). Systemic inflammation has been linked to depression and anxiety, two common mood disorders (Bahrami et al., 2019; Duivis et al., 2013).

The white blood cell count (WBC) is a nonspecific inflammatory marker that is usually measured as part of a complete blood count (CBC) panel. The quantitative measure of anisocytosis, red cell distribution width (RDW), is a simple, low‐cost parameter that is routinely reported as part of the CBC test (McPherson et al., 2021). Previous research has found a significant association between RDW and inflammatory markers such as hs‐CRP and erythrocyte sedimentation rate. This indicates that increased RDW may result from an underlying inflammatory state associated with negative outcomes (Kalay et al., 2011; Rawi et al., 2023).

Previous research has found that personal experiences with COVID‐19 diagnosis, mortality in acquaintances, and COVID‐19‐related stress are related to a significantly increased risk of emotional disorder symptomatology and that the COVID‐19 pandemic may increase demand for mental health services (Gallagher et al., 2020).

In contrast to measuring peripheral cytokine levels, studies on this topic have found that measuring peripheral blood cells, which are the source of some of these cytokines, by CBC is a cheap, easy, and rapid procedure. The neutrophil/lymphocyte ratio (NLR), which is determined by the ratio of these two key cells participating in the inflammatory response, has been accepted as a measure of the systemic inflammatory response (Khorasanchi et al., 2022; Zahorec, 2001). NLR has been reported to be higher in several psychiatric diseases, such as mood disorders, anxiety disorders, and schizophrenia, than in healthy adults, and has already been considered a biomarker of increased inflammatory response (Bustan et al., 2018; Ekinci & Ekinci, 2017). Platelet/lymphocyte ratio (PLR) is a biomarker used as a diagnostic of chronic inflammation, similar to NLR, and is useful in several disease groups, especially autoimmune illnesses and cancer (Erre et al., 2019; Kumarasamy et al., 2019) and is often used to diagnose psychiatric diseases (Özdin et al., 2017).

In this study, the main goal was to analyze the relationship between various blood inflammatory markers and psychological well‐being indicators in individuals diagnosed with COVID‐19. The specific inflammatory markers that were assessed included WBC, RDW, NLR, and PLR. The reason behind conducting this investigation was the observation of elevated inflammation levels. The psychological well‐being indicators that were examined encompassed depression, anxiety, stress, and quality of life in COVID‐19 patients. The aim of this study was to obtain a comprehensive understanding of how inflammation levels could potentially impact the mental well‐being of COVID‐19 patients.

## 2 MATERIALS AND METHODS

### 2.1 Study design

**FIGURE 1:**
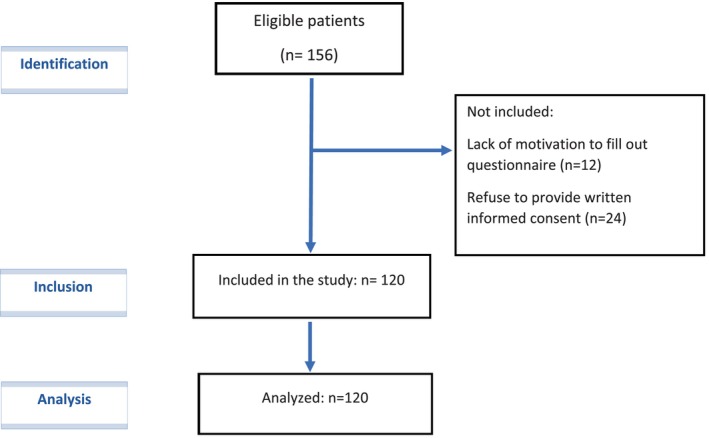
STROBE flow diagram of participants.

The current cross‐sectional study was carried out in 2021 within a sample of COVID‐19 patients from the Imam Reza Hospital in Mashhad, Iran. The total subjects included 120 COVID‐19 patients aged >30 years. The exclusion criteria were: cancer, autoimmune diseases, hepatic or renal failure, metabolic bone disease, and without antidepressant drug treatment during the previous 6 months. A written consent form was filled out by the participants. Diagram of the study participants showed in Figure 1.

### 2.2 General and clinical characteristics

Demographics and clinical characteristics including age, smoking status, and comorbidity data were collected from each participant at the baseline by trained interviewers.

### 2.3 Blood collection and biochemical measurements

Blood samples have been taken once from each patient and all of them were taken in the morning and after 12 h of midnight fasting. A CBC including red blood cell (RBC), hemoglobin (HGB), hematocrit (HCT), mean corpuscular volume (MCV), mean corpuscular hemoglobin (MCH) and mean corpuscular hemoglobin concentration (MCHC), platelet (PLT), platelet distribution width (PDW), mean platelet volume (MPV), RDW, and WBC were measured by using an auto hematology analyzer (Sysmex K‐800) for each individual.

### 2.4 Depression anxiety stress scales

The depression anxiety stress scale (DASS) is an accurate and valid tool to evaluate mood status (Henry & Crawford, 2005). This tool is a questionnaire that consists of three subscales including 7 questions, generally consists 21 items. Each question is ranged on a 4‐point (0–3) Likert scale to identify the severity of mood disorders including depression, anxiety, and stress. A score of each item must be doubled because DASS 21 is a brief version of DASS 42. In this tool, a higher score shows a higher degree of negative mood status and a lower score indicates a lower degree of negative emotion. The reliability and validity of DASS have been previously reported in the Iranian population (Sahebi et al., 2005). The scores for depression, anxiety, and stress were divided into two classifications: No or minimal scores, and some degree of mood disorder. Based on the scores obtained from each item were determined as follows: (≤9, No), (>9, some degree of depression), (≤7, no), (>7, some degree of anxiety), (≤14, no), (>4 some degree of stress).

### 2.5 Insomnia Severity Index (ISI)

The ISI is a seven‐item self‐report tool for determining insomnia symptoms and their consequences. The dimensions measured are the severity of sleep onset, sleep preservation, early morning awakening problems, sleep dissatisfaction, interference of sleep difficulties with daytime functioning, noticeability of sleep problems by others, and distress caused by sleep difficulties (Morin et al., 2011). Based on severity every item scored on a 0–4 scale. The total scale ranged from 0 to 28 and explained as follows: no insomnia (0–7), sub‐threshold insomnia (8–14), mild insomnia (15–21), and severe insomnia (22–28). The validity and reliability of the Persian version of this questionnaire have been confirmed in the Iranian population (Cronbach's a >0.8 and intra‐class correlation coefficient >0.7) (Yazdi et al., 2012).

### 2.6 Pittsburgh Sleep Quality Index (PSQI)

Sleep quality was evaluated using a 19‐item self‐reported PSQI questionnaire that assesses sleep quality over the last 30 days duration (Buysse et al., 1989). It consists of 19 items combined for 7 component scores, including subjective sleep quality, sleep latency, sleep duration, habitual sleep efficiency, sleep disturbances, use of sleep medication, and daytime dysfunction. The responses are scored on a 3‐point scale, ranging from 0 to 3. The overall score for sleep quality can be calculated by a combination of the 7 component scores, which range from 0 to 21. Patients were classified into two groups based on their PSQI score: the poor‐sleeper group (PSQI>5) and the good‐sleeper group (PSQI≤5). The Persian version of PSQI has also been validated in a previous study by Farrahi Moghaddam et al. in 2012 (Moghaddam et al., 2012).

### 2.7 Quality of life questionnaire

The Short Form Health Survey (SF‐36) validated questionnaire was used for evaluating the general quality of life. SF‐36 is categorized into eight subscales: Role Physical, Physical Functioning, General Health, Bodily Pain Social Functioning, Vitality, Role Emotional, and Mental Health. Scores of this questionnaire range from 0 to 100. The Persian version of SF‐36 was assessed in a previous study and indicated good reliability and construct validity (Montazeri et al., 2005).

### 2.8 Statistical analysis

SPSS Statistics for Windows v20 (SPSS, Inc., Chicago, IL) was used for statistical analysis. The normality of variables was analyzed using the Kolmogorov–Smirnov test. Descriptive statistics including mean and standard deviation (SD) were determined for all variables and expressed as mean ± SD for normally distributed variables and as the median and interquartile range (IQR) for non‐normally distributed variables. Moreover, categorical indices were demonstrated by number (%). Chi‐squared tests were used to compare the qualitative variables. Two independent sample *t*‐test and the Mann–Whitney test was used to evaluate the significant difference in normal and non‐normal variables between the two groups, respectively. Finally, multivariate linear regression was performed between hematological parameters and psychological scores. Five default assumptions were required to perform linear regression: (1) Linear relationship, (2) no or little multicollinearity (3) multivariate normality (4) homoscedasticity (5) no autocorrelation. These assumptions were checked, and then linear regression was performed. All analyses were considered bilateral, and a *p*‐Value of <0.05 was considered to be significant.

## 3 RESULTS

The demographic data of the patients are reported in Table 1, which shows the mean age, sex, smoking conditions, and underlying diseases of the participants. The number of patients participating in all subgroups of depression, anxiety, stress, sleep disorders, and quality of life questionnaire is listed separately.

**TABLE 1:** Demographics features of study subjects.

	Depression, *n* = 78	Anxiety, *n* = 90	Stress, *n* = 93	Sleep		Quality of life (score <55), *n* = 58
Variable				Insomnia, *n* = 28	Poor sleep quality, *n* = 85	
Gender						
Male	44 (57.8%)	49 (55.8%)	51(55.7%)	13 (45.5%)	42 (49.4%)	31 (53.4%)
Female	34 (42.2%)	41 (44.2%)	42(44.3%)	15 (54.5%)	43 (50.6%)	27 (46.6%)
Age (years)	60.42 ± 14.85	59.71 ± 14.33	60.41 ± 14.30	57.59 ± 14.18	61.42 ± 13.56	60.77 ± 15.56
Current smoking	18 (28.5%)	20 (26.3%)*	21 (27%)*	7 (25.0%)	16 (18.8%)	15 (25.8%)
Comorbidity						
Cardiovascular disease	14 (22.2%)	17 (22.4%)	17 (21.8%)	4 (14.2%)	14 (16.4%)	9 (15.5%)
Hypertension	25 (39.7%)	31 (40.8%)	32 (41.0%)	10 (35.7%)	30 (35.2%)	20 (34.4%)
Diabetes	25 (39.7%)	33 (43.4%)	35 (44.9%)	10 (35.7%)	32 (37.6%)	19 (32.7%)

*Note*: Data presented as mean ± SD or *n* (%). Obtained from *t‐test* for continuous variables and χ^2^ test for categorical variables.

* *p* < 0.05.

Table 2 shows the relationship between hematological parameters and variables of depression, anxiety, stress, sleep disorders, and quality of life. No blood parameters were significantly associated with depression, sleep disorders, and quality of life (*p* > 0.05), but anxiety score showed a significant association with MCHC (mean ± SD: 32.71 ± 1.68, *p* < 0.05), WBC (mean ± SD: 12.23 ± 5.43, *p* < 0.05) and PLR concentration (median (IQR): 28.72 (15.88–41.31), *p* < 0.05). In the stress subgroup, only RPR with a level of 0.07 (0.05–0.11) was associated with stress (*p* < 0.05).

**TABLE 2:** Hematological parameters in subjects with phycological disorders.

	Depression, *n* = 78	Anxiety, *n* = 90	Stress, *n* = 93	Sleep		Quality of life (score < 55), *n* = 58
Variable				Insomnia, *n* = 28	Poor sleep quality, *n* = 85	
RBC (10^12^/L)	4.58 ± 0.86	4.60 ± 0.84	4.60 ± 0.82	4.52 ± 0.78	4.69 ± 0.78	4.61 ± 0.79
Hemoglobin (g/dL)	12.81 ± 2.58	12.95 ± 2.43	12.94 ± 2.41	12.39 ± 2.37	13.13 ± 2.30	12.85 ± 2.38
Hematocrit	39.35 ± 6.59	39.92 ± 6.59	39.92 ± 6.51	38.09 ± 6.61	40.23 ± 6.16	39.59 ± 6.52
MCV (fL)	86.46 ± 6.77	87.06 ± 6.55	87.06 ± 6.56	87.13 ± 7.40	86.69 ± 6.43	87.06 ± 7.01
MCH (pg)	28.53 ± 2.68	28.68 ± 2.52	28.96 ± 2.49	28.30 ± 2.98	28.58 ± 2.51	28.54 ± 2.67
MCHC (g/dL)	32.83 ± 1.89	32.71 ± 1.68*	32.71 ± 1.65	32.54 ± 1.67	33.42 ± 6.72	33.54 ± 7.46
RDW (%)	14.75 ± 2.32	14.59 ± 1.99	14.67 ± 1.99	14.89 ± 2.05	14.48 ± 1.79	14.57 ± 1.82
PDW (fL)	14.02 ± 2.47	13.89 ± 2.41	13.88 ± 2.40	13.47 ± 2.80	13.83 ± 2.64	13.86 ± 2.48
WBC (10^3^/μL)	9.83 ± 4.72	12.23 ± 5.43*	11.25 ± 5.34	8.58 ± 3.55	9.26 ± 4.39	9.77 ± 4.76
MPV (fL)	9.70 ± 3.09	9.71 ± 2.77	9.72 ± 2.74	10.33 ± 0.87	9.78 ± 2.82	9.59 ± 3.41
NLR	8.15 (9.77)	7.64 (9.26)	7.86 (9.46)	7.47 (9.16)	7.75 (10.21)	7.35 (9.76)
RPR	0.07 (0.06)	0.07 (0.05)	0.07 (0.06)*	0.06 (0.04)	0.07 (0.05)	0.07 (0.08)
PLR	17.93 (18.39)	28.72 (25.43)*	23.25 (22.61)	17.20 (16.63)	16.1 (20.44)	13.57 (17.44)

*Note*: Data presented as mean ± SD or median (IQR). Obtained from independent sample *t*‐test for normal variable and Mann–Whitney test for the nonparametric variable.

Abbreviations: HCT, hematocrit; HGB, hemoglobin; MCH, mean corpuscular hemoglobin; MCHC, mean corpuscular hemoglobin concentration; MCV, mean corpuscular volume; NLR, neutrophil to lymphocyte ratio; PLR, platelet lymphocyte ratio; RBC, red blood cell; RDW, red cell distribution width; RPR, RDW to platelet count ratio; WBC, white blood cell.

* *p* < 0.05.

Multivariate linear regression analysis between hematological parameters and a score of mental health tests are indicated in Table 3. Linear regression indicated a positive relationship between RDW and PLR with depression (*β* = 0.086; *p* = 0.045 and *β* = 1.326; *p* = 0.016, respectively) and anxiety scores (*β* = 0.100; *p* = 0.038 and *β* = 1.356; *p* = 0.010, respectively). Moreover, a positive correlation was found between PLR and stress (β = 1.102; *p* = 0.012). Other hematological parameters were not significantly associated with depression, anxiety, stress, sleep disorders, and quality of life (*p* > 0.05) (Table 3).

**TABLE 3:** Multivariate linear regression between hematological parameters and psychological scores.

Hematological parameters	Depression		Anxiety		Stress		Insomnia		Sleep quality		Quality of life	
	β	*p*	β	*p*	β	*p*	β	*p*	β	*p*	β	*p*
RBC (10^12^/L)	−0.001	0957	−0.011	0.562	0.003	0.846	−0.224	0.245	0.150	0.498	0.009	0.951
Hemoglobin (g/dL)	−0.082	0.134	−0.079	0.165	−0.070	0.133	−0.742	0.198	0.083	0.900	0.356	0.440
Hematocrit	−0.136	0.355	−0.154	0.315	−0.105	0.397	−2.066	0.192	0.481	0.786	0.607	0.632
MCV (fL)	−0.197	0.147	−0.114	0.421	−0.207	0.070	0.646	0.658	−1.246	0.463	0.241	0.836
MCH (pg)	−0.100	0.051	−0.092	0.086	**−0.104**	**0.016**	−0.096	0.867	−0.264	0.687	0.198	0.664
MCHC (g/dL)	−0.037	0.764	−0.106	0.408	−0.074	0.480	−0.788	0.529	1.408	0.393	−0.734	0.462
RDW (%)	**0.086**	**0.045**	**0.100**	**0.038**	0.064	0.103	0.374	0.454	0.500	0.331	−0.144	0.717
PDW (fl)	0.084	0.121	0.014	0.798	0.046	0.299	−0.311	0.594	−0.118	0.873	−0.193	0.682
WBC (10^3^/μL)	0.021	0.841	−0.158	0.143	−0.093	0.296	−1.414	0.204	−0.865	0.475	−0.398	0.657
MPV (fL)	0.097	0.207	0.007	0.926	0.006	0.923	0.739	0.269	0.079	0.926	0.344	0.542
NLR	−0.375	0.058	−0.242	0.207	−0.219	0.168	−1.156	0.553	1.282	0.601	1.133	0.472
RPR	0.003	0.241	0.004	0.107	0.003	0.185	−0.017	0.535	−0.005	0.892	0.010	0.649
PLR	**1.326**	**0.016**	**1.356**	**0.010**	**1.102**	**0.012**	4.146	0.445	1.078	0.876	−3.372	0.440

*Note*: Adjusted for age, gender, and smoking status.

Abbreviations: HCT, hematocrit; HGB, hemoglobin; MCH, mean corpuscular hemoglobin; MCHC, mean corpuscular hemoglobin concentration; MCV, mean corpuscular volume; NLR, neutrophil to lymphocyte ratio; PLR, platelet lymphocyte ratio; RBC, red blood cell; RDW, red cell distribution width; RPR, RDW to platelet count ratio; WBC, white blood cell.

Bold values indicate significant association.

## 4 DISCUSSION

Our results suggest that higher depression and anxiety scores in COVID‐19 patients are associated with an enhanced inflammatory state, as assessed by higher hematological inflammatory markers including RDW and PLR. However, PLR was also associated with stress in participants.

Several theoretically viable hypotheses can be used to justify the potential prognostic role of anisocytosis in COVID‐19, including direct cytopathic injury disease associated with circulating erythrocytes or their bone marrow precursors, indirect erythrocyte damage caused by hemolytic anemia or intravascular coagulopathy, and profound iron metabolism disruption caused by the sustained inflammatory response (Henry et al., 2020). All of these factors would eventually lead to deranged erythrocyte biology and explain the wide range of erythrocyte sizes in circulation. The dramatic derangement of erythrocyte biology in patients with SARS‐CoV‐2 infection could also be explained by a coexisting indirect injury. First, cases of autoimmune hemolytic anemia have been linked to SARS‐CoV‐2 infection (Lazarian et al., 2020) a phenomenon that has been attributed to the high molecular similarity between the SARS‐CoV‐2 spike protein and the protein ankyrin 1 found on RBC surfaces (Angileri et al., 2020)In COVID‐19 patients who develop a severe or critical illness, intravascular coagulopathy, either localized to the lung parenchyma or disseminated, is also common (Lippi et al., 2020). The formation of micro‐ and microthrombi in various blood veins is a well‐known cause of erythrocyte injury, which would contribute to the existence of RBCs with numerous morphological defects in the circulation (Martinelli et al., 2020).

A higher WBC count has been identified in depressed and anxious people in several investigations (Abbaszadeh et al., 2021; Sunbul et al., 2016). One study showed a link between major depressive illness and leukocyte counts for males but not for women (Surtees et al., 2003). Also, WBC count was positively connected with anxiety score in women in another study (Pitsavos et al., 2006). In depressed patients, Darko et al. discovered leukocytosis, absolute neutrophilia, and relative lymphopenia. Also, the authors speculated that neutrophilia and leukocytosis could be side effects of taking specific medications (Darko et al., 1988). Several studies have demonstrated a link between depression and inflammation. Since WBC count is an independent predictor of cardiovascular disease, it is possible that higher WBC counts or the related inflammatory state could explain some of the elevated cardiac events shown in depressed people (Berk et al., 2013; Saharkhiz et al., 2021).

RDW has received a lot of attention in the last decade because of its ability to successfully predict the risk of death in the general population (Patel et al., 2010), patients with non‐cardiovascular critical illness, sepsis, pneumonia, and other respiratory tract infections (Hu et al., 2020; Luo et al., 2016). One study showed RDW to be a significant and independent predictor of disease severity and kidney injury in patients infected with SARS‐CoV‐2. In another study, patients with severe COVID‐19 had significantly higher RDW values than those with a milder form of the disease. Furthermore, both RDWs were found to be significant predictors of severe illness, with diagnostic accuracy ranging from 65% to 76% (Wang et al., 2020).

The PLR, a general inflammatory marker, represents a concurrent interaction of platelet and lymphocyte count, and it indicates aggregation as well as inflammatory pathways. It has been reported to be increased in response to a variety of acute and chronic proinflammatory conditions, and it has been linked to a poor prognosis in individuals with COPD and carcinomas (Erre et al., 2019; Li et al., 2018, 2020; Veronese et al., 2020). In COVID‐19 patients, the change in PLR from baseline appears to be linearly associated with the severity of illness and length of hospital stay (Qu et al., 2020). PLR appeared as an independent predictive factor for prolonged hospitalization in one study's multivariate analysis. A high PLR may indicate a more intense cytokine storm as a result of increased platelet activation (Fan, 2020). No major differences in PLR were seen in Tiwari's investigative process, as was observed in the current study. However, because platelets are a dynamic variable, the validity of PLR can only be determined by collecting follow‐up samples at different time points (Sahu et al., 2021). In spite of the strengths of this study, there are several limitations. The assessment of depression, anxiety, stress symptoms, sleep quality, insomnia, and quality of life was self‐administered based tools rather than more exact face‐to‐face interviews. Also, due to the condition of COVID‐19 wards in the first and second COVID‐19 peaks, researchers were instructed to limit their data collection time with the patients. Therefore, only necessary data were collected by the researchers. For instance, this study did not collect data on the patient's drug history. It will be worthwhile studying the relationship between hematological inflammatory markers and psychological function in COVID‐19 patients and also in COVID‐19 subjects that recovered from in a case–control study.

## 5 CONCLUSION

This study showed a positive association between depression/anxiety/stress symptoms and levels of hematological inflammatory markers including PLR and RDW in COVID‐19 patients, which persisted despite adjustment by potential confounders. Results of this study could help clinicians for patients with COVID‐19 who are affected by stress, anxiety, and depression.

## AUTHOR CONTRIBUTIONS

Majid Ghayour Mobarhan and Zahra Khorasanchi initially conceptualized and designed the study, Mohammad Rashidmayvan and Elahe Hasanzadeh upgraded the design. The manuscript was written by Kimia Mohammadhasani, Mohammad Vahedi Fard, and Payam Sharifan. Parisa Asadiyan‐Sohan was responsible for the design optimization and statistical analysis. Naiemeh Varaste, Mohammad Reza Shadmand Foumani Moghadam, and Nafise Afkhami contribute to sampling. Gordon Ferns performed English editing. All authors read and approved the final manuscript.

## FUNDING INFORMATION

The Mashhad University of Medical Sciences funded this study (grant nu: 981873).

## CONFLICT OF INTEREST STATEMENT

The authors declare that they have no conflict interests.

## ETHICS STATEMENT

The Ethics Committee of MUMS (Mashhad University of Medical Sciences), Mashhad, Iran, approved the trial (Ethic no: IR.MUMS.REC.1399.237).

## INFORMED CONSENT

Informed consent was obtained from all individual participant included in the study.

## Data Availability

The data are available on request.
